# Adherence to the 2018 World Cancer Research Fund/American Institute for Cancer Research cancer prevention recommendations and pancreatic cancer incidence and mortality: A prospective cohort study

**DOI:** 10.1002/cam4.3348

**Published:** 2020-07-27

**Authors:** Zhi‐Qing Zhang, Qu‐Jin Li, Fa‐Bao Hao, You‐Qi‐Le Wu, Shan Liu, Guo‐Chao Zhong

**Affiliations:** ^1^ Department of Hepatobiliary Surgery The Second Affiliated Hospital of Chongqing Medical University Chongqing China; ^2^ Department of Neurosurgery Qingdao Women and Children’s Hospital Qingdao University Qingdao China; ^3^ Department of Nutrition and Food Hygiene School of Public Health and Management, Chongqing Medical University Chongqing China; ^4^ Department of Pediatrics The People’s Hospital of Dazu District Chongqing China

**Keywords:** incidence, mortality, pancreatic cancer, WCRF/AICR

## Abstract

**Background:**

Whether adherence to the World Cancer Research Fund/American Institute for Cancer Research (WCRF/AICR) cancer prevention recommendations is associated with a reduced risk of pancreatic cancer remains controversial. Additionally, no study has investigated this association in the US population. Hence, we investigated the association of adherence to the 2018 WCRF/AICR cancer prevention recommendations with pancreatic cancer incidence and mortality in a US population.

**Methods:**

A population‐based cohort of 95 962 participants was identified. A score incorporating eight WCRF/AICR components was constructed to reflect adherence to the WCRF/AICR guidelines, with higher scores representing greater adherence to the guidelines. Cox and competing risk regression were used to calculate risk estimates for pancreatic cancer incidence and mortality, respectively. Restricted cubic spline functions were used to test nonlinearity.

**Results:**

In the fully adjusted model, higher overall WCRF/AICR scores were shown to be associated with lower risks of developing pancreatic cancer (hazard ratio_tertile 3 vs 1_:0.67; 95% confidence interval: 0.49, 0.90; *P*_trend_ = .0099) and mortality due to this cancer (subdistribution hazard ratio_tertile 3 vs 1_ 0.65; 95% confidence interval: 0.47, 0.89; *P*_trend_ = .0108) in a linear dose–response manner (all *P*_nonlinearity_ > .05). The component “be physically active” was shown to be a key contributor to the observed associations. No association of the diet‐specific WCRF/AICR score with pancreatic incidence and mortality was found.

**Conclusions:**

Adherence to the 2018 WCRF/AICR guidelines, especially “be physically active,” confers reduced risks of pancreatic cancer incidence and mortality in the US population; however, adherence to dietary components alone does not confer such beneficial effects.

## Graphical abstract

In this prospective study of 95962 American adults, we found that greater adherence to the 2018 WCRF/AICR recommendations was associated with lower risks of pancreatic cancer incidence and mortality. However, such beneficial effects were not found for adherence to diet‐specific recommendations alone.Importantly, the component “be physically active” was found to be a key driver of the observed associations.

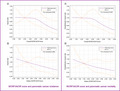

## 1 INTRODUCTION

In the United States, the incidence of pancreatic cancer is relatively low, but it is the fourth leading cause of cancer‐associated death, with an estimated 57 600 cases and 47 050 deaths in 2020.^1^ The exact mechanisms for pancreatic carcinogenesis remain unclear, but lifestyle factors have been suggested to play a critical role.^2^, ^3^ Although numerous observational studies have investigated the association between individual lifestyle factors and pancreatic cancer risk,^3^ they fail to consider the potential interactions among these factors. Hence, investigating the association between lifestyle patterns (eg, Mediterranean diet), in which various factors are integrated into an entirety, and pancreatic cancer risk is essential for understanding the role of lifestyle behaviors in the etiology of this cancer.^4^

The World Cancer Research Fund/American Institute for Cancer Research (WCRF/AICR) proposed an updated set of 10 recommendations for cancer prevention in 2018.^5^ Compared to their 2007 report,^6^ the 2018 report has shifted the emphasis of recommendations toward a more holistic focus and removed the recommendation associated with salt as well as moldy grains or legumes.^7^ Adherence to the 2007 and 2018 WCRF/AICR recommendations has been shown to be associated with decreased risks of breast and colorectal cancers.^8^, ^9^, ^10^ To date, two studies have investigated the association between adherence to the 2007 WCRF/AICR recommendations and pancreatic cancer risk in the European population, but presented inconsistent results.^11^, ^12^ Specifically, a 2012 prospective study on this topic revealed a null association,^12^ whereas a subsequent case‐control study observed that adherence to the WCRF/AICR recommendations conferred a reduced risk of developing pancreatic cancer.^11^

To our knowledge, no study has explored this topic in the US population; moreover, whether adherence to the latest WCRF/AICR recommendations confers a reduced pancreatic cancer risk has not been determined. Hence, we performed a prospective study to examine the hypothesis that adherence to the 2018 WCRF/AICR recommendations is associated with reduced risks of pancreatic cancer incidence and mortality in a population‐based cohort of 95 962 American adults.

## 2 METHODS

### 2.1 Study population

Around 155 000 subjects aged 55‐74 years were enrolled to the Prostate, Lung, Colorectal, and Ovarian (PLCO) Cancer Screening Trial between 1993 and 2001. This trial is a large randomized clinical study designed to investigate the potential beneficial effects of selected screening tests on the risk of mortality from prostate, lung, colorectal, and ovarian cancers. Design and implementation of the PLCO Cancer Screening Trial have been reported in the literature.^13^ All subjects gave their written informed consents and the trial was approved by the Institutional Review Boards at 10 screening centers and the US National Cancer Institute.

**FIGURE 1:**
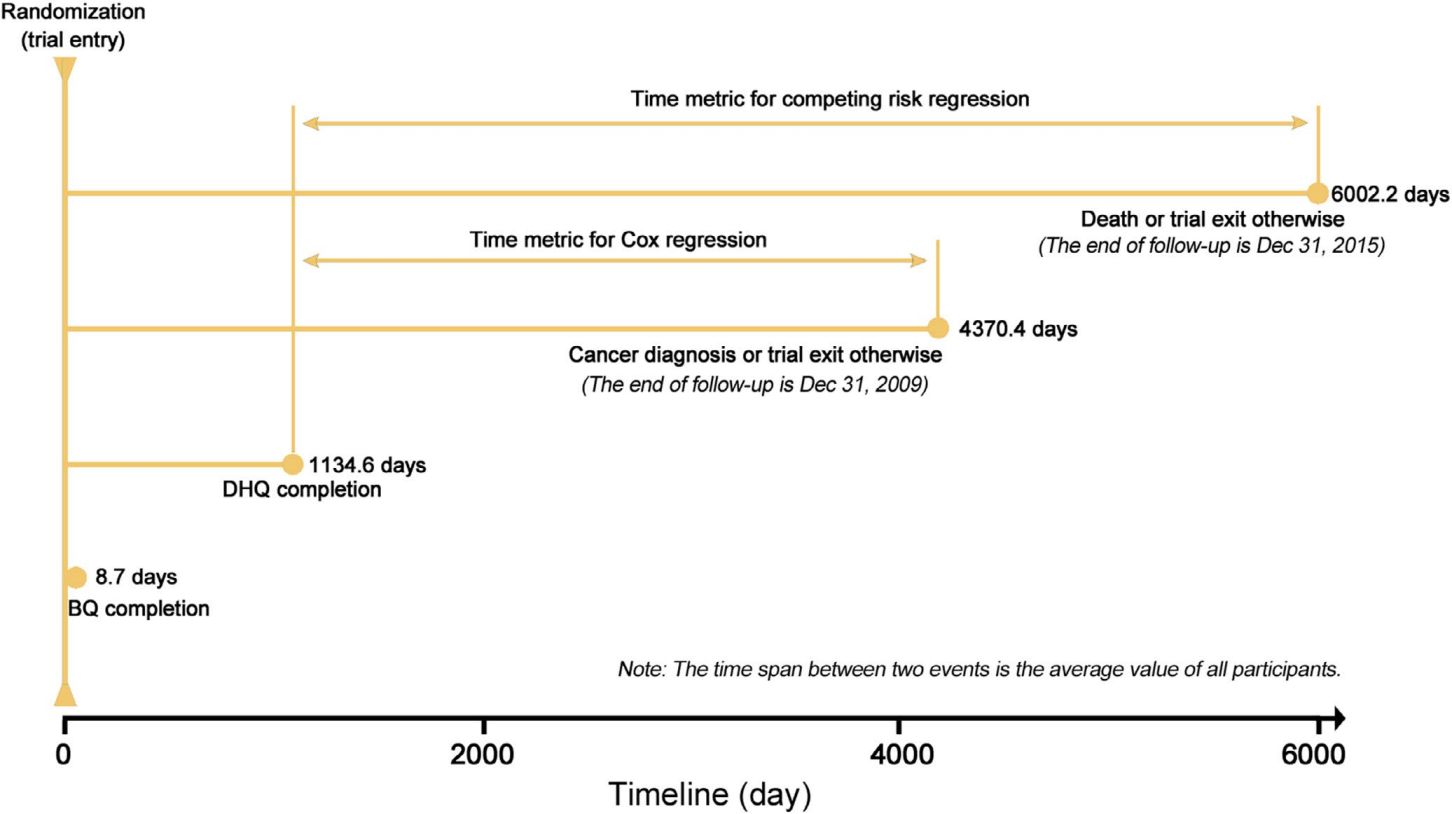
The timeline and follow‐up scheme of our study. DHQ, diet history questionnaire; BQ, baseline questionnaire

The subjects with any of the following conditions were not eligible for the present analysis: (a) subjects who did not finish a diet history questionnaire (DHQ; n = 36 076); (b) subjects who returned an invalid DHQ, which refers to DHQ completion date prior to death date, missing DHQ completion date, ≥8 missing DHQ items, and extreme calorie intakes (>99th percentile or <1th percentile) (n = 5364); (c) subjects who were diagnosed with any cancer before trial entry or DHQ completion (n = 9684); (d) subjects who did not finish or return a baseline questionnaire (n = 7675); (e) subjects who were diagnosed with primary adenocarcinoma of the endocrine pancreas during the study period (n = 17); (f) subjects with outcome events (ie, incident pancreatic cancer or death) occurred between trial entry and DHQ completion (n = 70); and (g) subjects whose pancreatic cancer was not the first diagnosed cancer (n = 39). After exclusions, a total of 95 962 subjects were eligible for this analysis. All participants were followed up from DHQ completion to the occurrence of outcome events, study dropout, or the end of follow‐up, whichever occurred first (Figure 1). In this analysis, follow‐up ended on December 31, 2009, for cancer incidence and December 31, 2015, for cancer mortality.

### 2.2 Data collection

Age, energy intake from diet, alcohol intake, and daily food consumption were collected with the DHQ, a 137‐item food frequency questionnaire used for evaluating the consumption of foods and supplements during the past 12 months. The validity of the DHQ has been confirmed previously.^14^ Daily food consumption was calculated by multiplying food frequency by portion size. The frequency and duration of moderate and vigorous activities and breastfeeding duration were collected through a self‐administrated supplemental questionnaire. In our study, the physical activity level refers to the total time of moderate to vigorous activity per week. Remaining information, including gender, ethnicity, body mass index (BMI), aspirin use, cigarette smoking, personal history of diabetes, and family history of pancreatic cancer, was collected with a sex‐specific baseline questionnaire.

### 2.3 WCRF/AICR score construction

We constructed a score to quantify adherence to the 2018 WCRF/AICR guidelines following a standardized scoring system described in the literature.^15^ Of 10 recommendations from the 2018 WCRF/AICR guideline,^5^ the recommendation about supplements was not considered, as we could not ascertain a specific reason for supplement use; the recommendation for cancer survivors was also not considered, as our target population was set as the general population. For the recommendation to “be at a healthy weight,” the waist circumference was not included, as this variable was not collected in the PLCO Cancer Screening Trial.

The score assigned to each recommendation was 0 points for non‐adherence, 0.5 points for partial adherence, and 1 point for full adherence. For the recommendation to “eat a diet high in whole grains, vegetables, fruit, and beans,” which consists of two sub‐recommendations, the score was computed as the mean of scores assigned to each sub‐recommendation. All scores were then summed to calculate an individual's overall WCRF/AICR score that ranges from 0 to 8 points. Higher scores represent greater adherence to the WCRF/AICR guidelines. We also computed a dietary WCRF/AICR score by summing the scores for five recommendations on diet. Table S1 shows the construction of the WCRF/AICR score in detail.

### 2.4 Outcome ascertainment

Pancreatic cancer was confirmed via a mailed annual study update form that required participants to answer whether they received a diagnosis of any cancer, the date, and location of cancer diagnosis, and the type of cancer. Participants failing to return the form were contacted repeatedly via e‐mail or telephone. Family reports and death certificates were used as supplemental sources for case ascertainment. Relevant medical records were scrutinized for further confirmation of pancreatic cancer cases. Pancreatic cancer referred to primary adenocarcinoma of the exocrine pancreas only (ICD‐O‐2 code: C25.0‐C25.3, C25.7‐C25.9) in this analysis. The information on death was obtained from the annual study update form and was adjudicated through periodic linkage with the National Death Index for improving its completeness. The underlying causes of death were determined by death certificates.

### 2.5 Statistical analysis

As the variables “physical activity level” and “breastfeeding during” had 24 229 and 10 101 missing values, respectively, for increasing statistical power and reducing selection bias, we used multiple imputations with chained equations to impute missing data (the number of imputations = 25).^16^ The following variables were used to produce imputed data sets^17^: age, gender, ethnicity, BMI, energy intake from diet, educational degree, aspirin use, cigarette smoking, alcohol consumption, personal history of diabetes, physical activity level, family history of pancreatic cancer, breastfeeding during, and pancreatic cancer incidence or mortality. All primary analyses were repeated in subjects with complete data for comparison.

Cox proportional hazards regression was performed to estimate hazard ratios (HRs) and 95% confidence intervals (CIs) for pancreatic cancer incidence, with person‐year as the time variable. We did not find any evidence for the violation of proportional hazards assumption using the Schoenfeld residuals method (all *P* > .05). For minimizing the possible influences of competing risk bias on the relevant results, we used competing risk regression to estimate subdistribution HRs (SHRs) and 95% CIs for pancreatic cancer mortality, with non‐pancreatic cancer deaths as competing events.^18^ In regression analyses, overall and dietary WCRF/AICR scores were classified into tertiles, and the lowest tertile was used as the reference group. For the purpose of determining a linear trend across tertiles of the WCRF/AICR score, we first assigned the median of each tertile to each individual in the tertile and then regarded it as a continuous variable in Cox regression. Additionally, the WCRF/AICR score was modeled as a continuous variable directly to calculate risk estimates and 95% CIs per 1‐point increment.

We determined the covariate included in multivariable analyses on the basis of the change‐in‐estimate strategy^19^ and the literature review. In this analysis, model 1 was adjusted for the variables determined by the change‐in‐estimate strategy; model 2 was adjusted for age, gender, ethnicity, educational degree, personal history of diabetes, cigarette smoking, aspirin use, family history of pancreatic cancer, and energy intake from diet. For the associations of dietary WCRF/AICR score with the incidence of and mortality from pancreatic cancer, model 2 was additionally adjusted for BMI and physical activity level. We calculated the *E*‐value to evaluate how robust the observed associations of adherence to the WCRF/AICR score with pancreatic cancer incidence and mortality were to the potential residual confounding.^20^ This measure represents what the minimum risk estimates would have to be for an unmeasured confounder to explain away the observed association, conditional on the measured covariates.

We used restricted cubic spline models^21^ with three knots located at the 10th, 50th, and 90th percentiles to provide a thorough description for pancreatic cancer incidence and mortality across the full range of WCRF/AICR score. For comparison with the results from Cox regression, we set the median of the first tertile of WCRF/AICR score as the referent (ie, the referents were 2.00 and 1.50 for overall and dietary WCRF/AICR scores, respectively). A *P*_nonlinearity_ was estimated by examining the null hypothesis that the estimate value of the second spline is equal to 0.^21^ Predefined subgroup analyses were performed to investigate whether the observed associations were modified by age (≥65 vs <65 years), gender (men vs women), trial arm (intervention vs control), current smoking (yes vs no), aspirin use (yes vs no), and energy intake from diet (≥median vs <median). A *P*_interaction_ was obtained through a likelihood ratio test, in which models with and without interaction terms were compared. Two sensitivity analyses were performed to evaluate the stability of our results: excluding pancreatic cancer cases or deaths observed within the first two years of follow‐up and excluding participants with extreme energy intakes (ie, <800 or >4000 kcal/day for men and <500 or >3500 kcal/day for women^22^). Additionally, we assessed the association by the individual components and ignored a single component, in turn, to determine their relative importance in the overall WCRF/AICR score.

Statistical analyses were conducted with STATA version 12.0 (StataCorp, College Station, TX). The results were considered statistically significant when a two‐tailed *P* value was less than .05.

## 3 RESULTS

### 3.1 Participant characteristics

The median (range) of overall and dietary WCRF/AICR scores was 4.25 (0.25‐8.00) and 3.00 (0.25‐5.00), respectively. Participants in the highest vs the lowest tertiles of the overall WCRF/AICR score were less likely to be male, non‐Hispanic white, aspirin user, current smoker, and have a personal history of diabetes, and had a higher educational degree (Table 1). Moreover, participants in the highest tertile of the overall WCRF/AICR score had lower BMI, alcohol consumption, and energy intake from diet, and higher physical activity level than those in the lowest tertile.

**TABLE 1:** Baseline characteristics of the study population according to the overall WCRF/AICR score

Characteristics^a^	Overall	Tertiles of the overall WCRF/AICR score		
		0.25‐3.75	4.00‐4.75	5.00=8.00
Number of participants	95 962	29 532	33 339	33 091
Age (yeas)	65.4 ± 5.7	64.9 ± 5.6	65.6 ± 5.7	65.8 ± 5.8
Male	46 747 (48.7)	20 705 (70.1)	17 331 (52.0)	8711 (26.3)
Body mass index (kg/m^2^)	27.2 ± 4.8	29.6 ± 4.9	27.2 ± 4.5	25.1 ± 3.9
Energy intake from diet (kcal/day)	1742.6 ± 736.0	2046.5 ± 827.1	1717.6 ± 713.9	1496.5 ± 550.7
Alcohol consumption (g/day)	9.8 ± 25.7	15.9 ± 35.5	9.2 ± 23.8	5.0 ± 12.6
Physical activity level (min/week)^b^	119.7 ± 127.3	61.9 ± 93.6	115.8 ± 124.2	175.2 ± 132.6
Trial arm				
Intervention	49 268 (51.3)	15 350 (52.0)	17 016 (51.0)	16 902 (51.1)
Control	46 694 (48.7)	14 182 (48.0)	16 323 (49.0)	16 189 (48.9)
Race				
Non‐Hispanic white	87 517 (91.2)	27 193 (92.1)	30 339 (91.0)	29 985 (90.6)
Non‐Hispanic black	3050 (3.2)	905 (3.1)	1067 (3.2)	1078 (3.3)
Hispanic	1400 (1.4)	395 (1.3)	513 (1.5)	492 (1.5)
Others ^c^	3995 (4.2)	1039 (3.5)	1420 (4.3)	1536 (4.6)
Educational degree				
College below	60 908 (63.5)	19 730 (66.8)	21 107 (63.3)	20 071 (60.7)
College graduate	16 990 (17.7)	4907 (16.6)	5925 (17.8)	6158 (18.6)
Postgraduate	18 064 (18.8)	4895 (16.6)	6307 (18.9)	6862 (20.7)
Aspirin use				
Yes	45 278 (47.2)	14 979 (50.7)	15 961 (47.9)	14 338 (43.3)
No	50 684 (52.8)	14 553 (49.3)	17 378 (52.1)	18 753 (56.7)
Smoking status				
Current	8906 (9.3)	3661 (12.4)	3165 (9.5)	2080 (6.3)
Former	41 684 (43.4)	15 153 (51.3)	14 772 (44.3)	11 759 (35.5)
Never	45 372 (47.3)	10 718 (36.3)	15 402 (46.2)	19 252 (58.2)
History of diabetes				
Yes	6304 (6.6)	2883 (9.8)	2054 (6.2)	1367 (4.1)
No	89 658 (93.4)	26 649 (90.2)	31 285 (93.8)	31 724 (95.9)
Family history of pancreatic cancer				
Yes	2476 (2.6)	662 (2.2)	902 (2.7)	912 (2.8)
No	91 025 (94.8)	27 997 (94.8)	31 581 (94.7)	31 447 (95.0)
Possibly	2461 (2.6)	873 (3.0)	856 (2.6)	732 (2.2)

Abbreviation: WCRF/AICR, World Cancer Research Fund and American Institute for Cancer Research.

^a^ Data are mean (standard deviation) or number (percentage) as indicated.

^b^ Total time of moderate to vigorous activity per week.

^c^ “Others” refers to Asian, Pacific Islander, or American Indian.

### 3.2 WCRF/AICR score and pancreatic cancer incideNCE

During a mean (± standard deviation) follow‐up of 8.87 (± 1.91) years (850 730.7 person‐years), a total of 337 pancreatic cancer cases were documented. In the fully adjusted model, participants in the highest vs the lowest tertiles of the overall WCRF/AICR score had a lower risk of pancreatic cancer incidence (HR_tertile 3 vs 1_:0.67; 95% CI: 0.49, 0.90; *P*_trend_ = .0099; *E*‐value: 2.35) (Table 2). However, such a beneficial effect was not found for the dietary WCRF/AICR score (Table 2). When the above analyses were conducted in participants with complete data, similar results were observed (Table S2).

**TABLE 2:** Hazard ratios of the association between WCRF/AICR score and pancreatic cancer incidence

Tertiles of WCRF/AICR score (range)	Number of cases	Person‐years	Incidence/10000 person‐years	Hazard ratio (95% confidence interval)		
				Unadjusted	Model 1^a^	Model 2 ^b^
Overall WCRF/AICR score						
Tertile 1 (0.25‐3.75)	125	258 405.0	4.84	1.00 (reference)	1.00 (reference)	1.00 (reference)
Tertile 2 (4.00‐4.75)	123	295 383.5	4.16	0.86 (0.67, 1.10)	0.87 (0.68, 1.13)	0.87 (0.67, 1.13)
Tertile 3 (5.00‐8.00)	89	296 942.2	3.00	0.62 (0.47, 0.81)	0.67 (0.50, 0.90)	0.67 (0.49, 0.90)
*P _trend_*				.0006	.0100	.0099
Continuous (1‐ponit increment)	337	850 730.7	3.96	0.85 (0.77, 0.94)	0.88 (0.78, 0.99)	0.88 (0.78, 0.99)
Dietary WCRF/AICR score						
Tertile 1 (0.25‐2.75)	106	252 216.7	4.20	1.00 (reference)	1.00 (reference)	1.00 (reference)
Tertile 2 (3.00‐3.25)	102	232 606.9	4.39	1.04 (0.80, 1.37)	1.04 (0.78, 1.37)	1.04 (0.78, 1.38)
Tertile 3 (3.50‐5.00)	129	365 907.1	3.53	0.84 (0.65, 1.09)	0.83 (0.62, 1.11)	0.83 (0.63, 1.11)
*P _trend_*				.1282	.1444	.1510
Continuous (1‐ponit increment)	337	850 730.7	3.96	0.88 (0.75, 1.03)	0.86 (0.72, 1.03)	0.86 (0.72, 1.03)

Abbreviation: WCRF/AICR, World Cancer Research Fund and American Institute for Cancer Research.

^a^ Adjusted for variables determined by change‐in‐estimate strategy, namely age (years), sex (male, female), smoking status (current, former, never), history of diabetes (yes, no), and total energy intake (kcal/day).

^b^ Adjusted for age (years), sex (male, female), race (non‐Hispanic white, non‐Hispanic black, Hispanic, others), educational degree (college below, college graduate, postgraduate), smoking status (current, former, never), aspirin use (yes, no), history of diabetes (yes, no), family history of pancreatic cancer (yes, no), and energy intake from diet (kcal/day). For the association of dietary WCRF/AICR score with pancreatic cancer incidence, model 2 was further adjusted for body mass index (kg/m^2^) and physical activity level (min/week).

**FIGURE 2:**
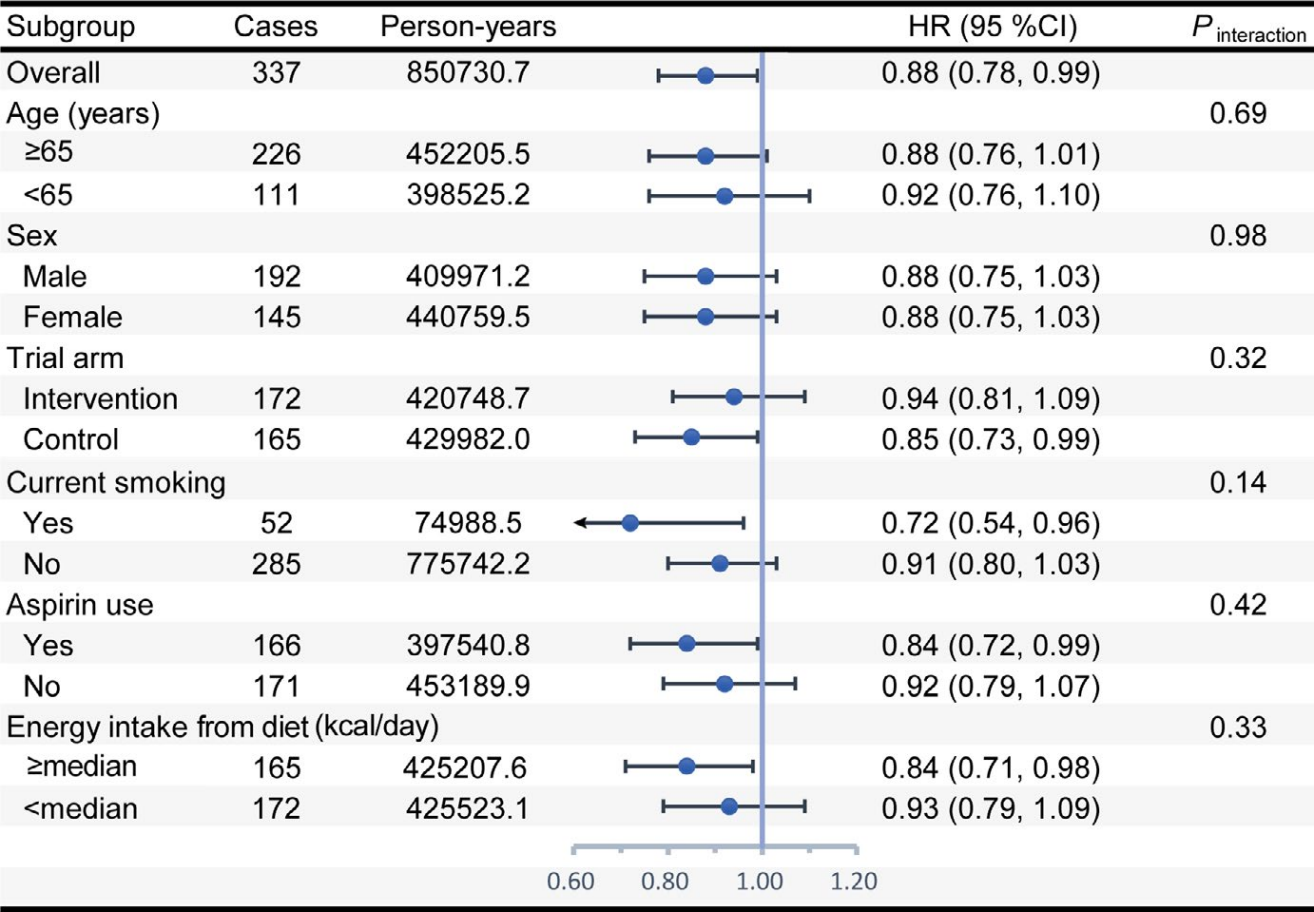
Subgroup analyses on the association of the overall WCRF/AICR score with pancreatic cancer incidence. HR represents risk estimate per 1‐point increment in the overall WCRF/AICR score and was adjusted for age, sex, race, educational degree, smoking status, aspirin use, history of diabetes, family history of pancreatic cancer, and energy intake from diet. In each case, HR was not adjusted for the stratification factor. *P*_interaction_ was calculated from a likelihood ratio test. The median value of energy intake from diet was equal to 1612.2. WCRF/AICR, World Cancer Research Fund/American Institute for Cancer Research; HR, hazard ratio; CI, confidence interval

Restricted cubic spline models revealed a significant inverse linear dose–response relationship for overall but not dietary WCRF/AICR scores (Figure S1). The observed association of the overall WCRF/AICR score with pancreatic cancer incidence was not modified by predefined stratification factors (all *P*_interaction_ > .05) (Figure 2). The initial associations did not alter substantially after excluding pancreatic cancer cases observed within the first two years of follow‐up (n = 58) or participants with extreme energy intakes (n = 2684) (Table S3). Of eight components of the overall WCRF/AICR score, only adherence to “be physically active” was found to be associated with a reduced risk of pancreatic cancer incidence (*P*_trend_ = .0377) (Table S4). After excluding “be physically active” or “limit consumption of sugar‐sweetened drinks,” adherence to the WCRF/AICR recommendations no longer conferred a reduced risk of pancreatic cancer incidence (Figure S2).

### 3.3 WCRF/AICR score and pancreatic cancer mortality

We documented 307 pancreatic cancer deaths over an average follow‐up of 13.34 ± 3.43 years (1 279 748.5 person‐years). The highest vs the lowest tertiles of the overall WCRF/AICR score was found to be associated with a lower risk of pancreatic cancer mortality (the maximally adjusted SHR_tertile 3 vs 1_ 0.65; 95% CI 0.47, 0.89; *P*_trend_ = .0108; *E*‐value: 2.45) (Table 3). However, no significant association was observed for the dietary WCRF/AICR score (Table 3). We obtained similar results when conducting the above analyses in participants with complete data (Table S5).

**TABLE 3:** Subdistribution hazard ratios of the association between WCRF/AICR score and pancreatic cancer mortality

Tertiles of WCRF/AICR score (range)	Number of deaths	Person‐years	Mortality/10 000 person‐years	Subdistribution hazard ratio (95% confidence interval)		
				Unadjusted	Model 1^a^	Model 2 ^b^
Overall WCRF/AICR score						
Tertile 1 (0.25‐3.75)	112	384 861.0	2.91	1.00 (reference)	1.00 (reference)	1.00 (reference)
Tertile 2 (4.00‐4.75)	116	443 775.4	2.61	0.91 (0.70, 1.18)	0.92 (0.70, 1.20)	0.92 (0.70, 1.20)
Tertile 3 (5.00‐8.00)	79	451 112.0	1.75	0.61 (0.46, 0.82)	0.65 (0.47, 0.89)	0.65 (0.47, 0.89)
*P _trend_*				0.0011	.0107	.0108
Continuous (1‐ponit increment)	307	1 279 748.5	2.40	0.86 (0.77, 0.95)	0.88 (0.78, 0.99)	0.88 (0.78, 0.99)
Dietary WCRF/AICR score						
Tertile 1 (0.25‐2.75)	95	379 726.3	2.50	1.00 (reference)	1.00 (reference)	1.00 (reference)
Tertile 2 (3.00‐3.25)	93	350 113.9	2.66	1.06 (0.80, 1.42)	1.05 (0.78, 1.42)	1.05 (0.78, 1.42)
Tertile 3 (3.50‐5.00)	119	549 908.4	2.16	0.86 (0.66, 1.13)	0.85 (0.63, 1.15)	0.85 (0.63, 1.14)
*P _trend_*				0.2075	.2011	.1941
Continuous (1‐ponit increment)	307	1 279 748.5	2.40	0.88 (0.75, 1.04)	0.86 (0.71, 1.04)	0.86 (0.71, 1.03)

Abbreviation: WCRF/AICR, World Cancer Research Fund and American Institute for Cancer Research.

^a^ Adjusted for variables determined by change‐in‐estimate strategy, namely age (years), sex (male, female), smoking status (current, former, never), history of diabetes (yes, no), and total energy intake (kcal/day).

^b^ Adjusted for age (years), sex (male, female), race (non‐Hispanic white, non‐Hispanic black, Hispanic, others), educational level (college below, college graduate, postgraduate), smoking status (current, former, never), aspirin use (yes, no), history of diabetes (yes, no), family history of pancreatic cancer (yes, no), and energy intake from diet (kcal/day). For the association of dietary WCRF/AICR score with pancreatic cancer mortality, model 2 was further adjusted for body mass index (kg/m^2^) and physical activity level (min/week).

**FIGURE 3:**
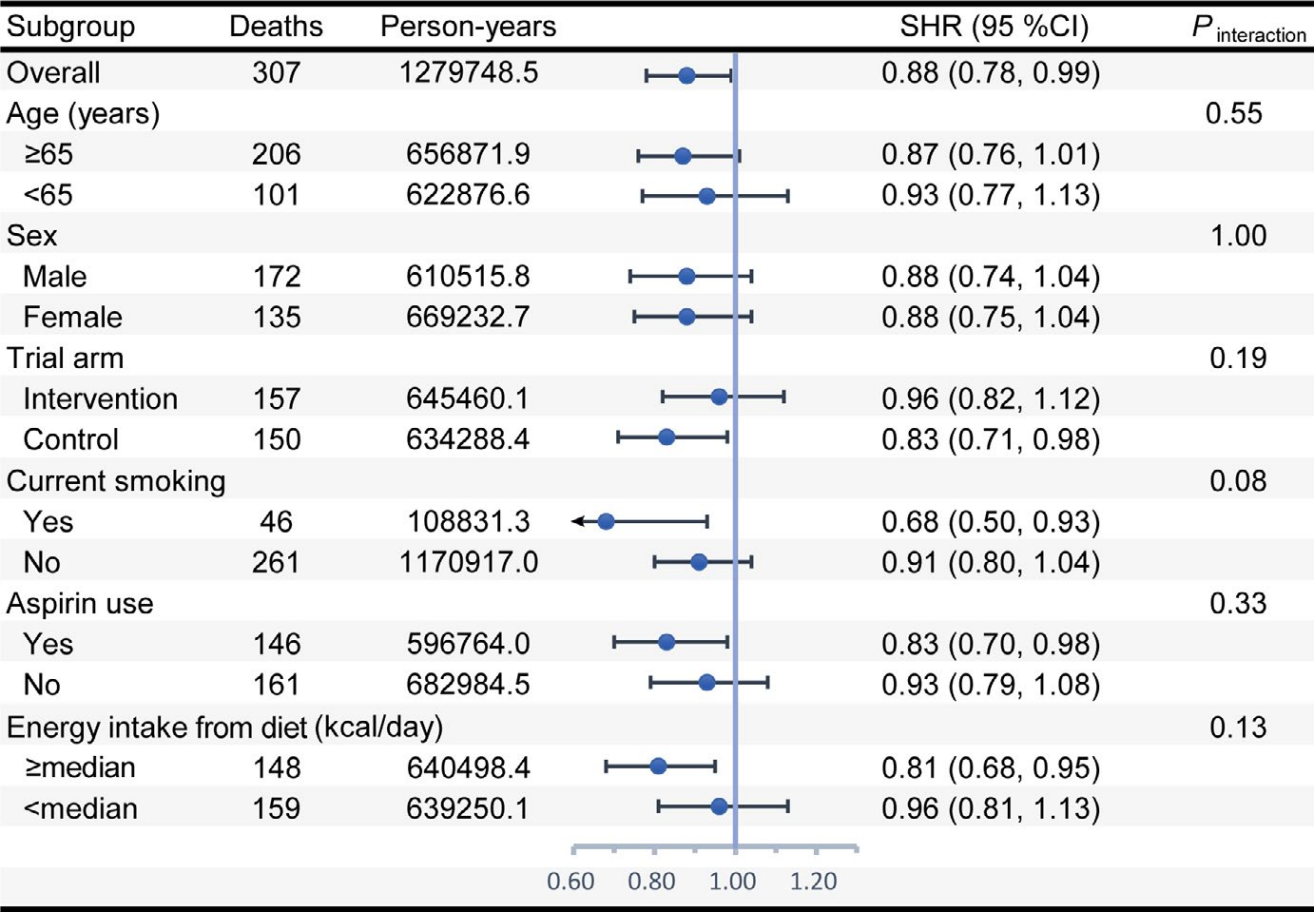
Subgroup analyses on the association of the overall WCRF/AICR score with pancreatic cancer mortality. SHR represents risk estimate per 1‐point increment in the overall WCRF/AICR score and was adjusted for age, sex, race, educational degree, smoking status, aspirin use, history of diabetes, family history of pancreatic cancer, and energy intake from diet. In each case, SHR was not adjusted for the stratification factor. *P*_interaction_ was calculated from a likelihood ratio test. The median value of energy intake from diet was equal to 1612.2. WCRF/AICR, World Cancer Research Fund/American Institute for Cancer Research; SHR, subdistribution hazard ratio; CI, confidence interval

A significant inverse linear dose–response relationship to the risk of death from pancreatic cancer was found for overall but not dietary WCRF/AICR scores (Figure S3). The observed association between the overall WCRF/AICR score and pancreatic cancer mortality was not modified by age, sex, trial arm, current smoking, aspirin use, and energy intake from diet (all *P*_interaction_ > .05) (Figure 3). The primary association persisted in sensitivity analyses (Table S6). Of eight recommendations, only adherence to the recommendation to “be physically active” was found to be associated with a decreased risk of death from pancreatic cancer (*P*_trend_ = .0543) (Table S7). Excluding “be physically active” or “limit consumption of sugar‐sweetened drinks” resulted in a non‐significant association of the overall WCRF/AICR score with pancreatic cancer mortality (Figure S4).

## 4 DISCUSSION

In this prospective study of 95 962 participants, we found that greater adherence to the 2018 WCRF/AICR recommendations, as reflected by the higher overall WCRF/AICR scores, conferred lower risks of pancreatic cancer incidence and mortality. However, such beneficial effects were not found for adherence to dietary recommendations.

The diet has been perceived to play a critical role in the etiology of pancreatic cancer for a long‐term period.^4^, ^23^ A systematic review found that better diet quality conferred a decreased risk of developing pancreatic cancer,^24^ which is similar to the findings from a meta‐analysis.^25^ Our previous work in this area also showed that having a diet high in antioxidants might be favorably associated with pancreatic cancer incidence.^26^ However, in this analysis, we did not find the expected beneficial effects of adherence to the WCRF/AICR dietary recommendations on risks of pancreatic cancer incidence and mortality, suggesting potential invalidity of these recommendations for pancreatic cancer prevention among the US population. In fact, similar to our findings, a recent large prospective study found that adherence to a Mediterranean diet was not associated with the risk of pancreatic cancer.^27^ The specific reasons for the inconsistency on the role of diet in the etiology of pancreatic cancer in the literature are unclear and possibly result from the differences in the study population, study design and methodology, dietary patterns under investigation, or some combinations of these factors. Collectively, more studies are needed to understand the role of dietary habits in the pathogenesis of pancreatic cancer.

Currently, whether physical activity is protective against pancreatic cancer remains to be elucidated.^28^, ^29^, ^30^ In this study, we used moderate to vigorous activity time as an indicator of physical activity level and found that adherence to the recommendation to “be physically active” conferred reduced risks of pancreatic cancer incidence and mortality; moreover, significant inverse associations with the overall WCRF/AICR score were no longer observed after the exclusion of the component “be physically active.” These facts suggest that the WCRF/AICR recommendation on physical activity could be a key driver of the observed associations and highlight the importance of keeping physically active for reducing the risk of pancreatic cancer.

Interestingly, our study observed a null association of adherence to the component “be a healthy weight” with the risk of pancreatic cancer. To explain this observation is challenging, considering that higher body fatness has been regarded as a convincing cause of pancreatic cancer in the 2018 WCRF/AICR report.^31^ A recent follow‐up study found that the positive association of BMI with the risk of pancreatic cancer was more pronounced in participants aged 30‐49 years (HR per 5‐unit increase in BMI: 1.25; 95% CI: 1.18, 1.33) than in those aged 70‐89 years (HR per 5‐unit increase in BMI: 1.13; 95% CI: 1.02, 1.26)^32^; also, another follow‐up study found that there was no association between obesity and the risk of pancreatic cancer in older women (mean age = 61.6 years).^33^ Considering the fact that the mean age of participants at baseline was 65.4 years in our study along with the results from the above‐mentioned two studies, one straightforward explanation for the null association we observed is the lack of an association of body fatness with the risk of pancreatic cancer in nature in this older population. In fact, a prospective study of 51 251 Singaporean Chinese also revealed a null association between BMI and the risk of pancreatic cancer in never smokers.^34^ An alternative explanation for the observed null association is that different measures of body fatness have different associations with the risk of pancreatic cancer. Indeed, in the European Prospective Investigation into Cancer and Nutrition study (EPIC), waist circumference but not BMI was found to be positively associated with the risk of pancreatic cancer,^35^ indicating that the waist circumference could be a better indicator of body fatness than BMI.^36^ However, as waist circumference was not available in the PLCO Cancer Screening Trial, we did not consider this measure when developing the WCRF/AICR scoring system, which possibly results in the observed null association.

Previously, the EPIC study found that adherence to the 2007 WCRF/AICR guidelines was not associated with a reduced risk of developing pancreatic cancer (HR_quartile 4 vs 1_:1.00; 95% CI: 0.78, 1.28; *P*_trend_ = .684),^12^ which is inconsistent with our findings. Remarkably, the EPIC study documented up to 783 pancreatic cancer cases during the follow‐up period,^12^ suggesting that its null findings might not be due to the limited cases. Instead, the observed inconsistency may be due to the difference in the study population. For example, the EPIC study was conducted in 386 355 European adults with a mean age of 53.0 years, whereas our study was conducted in 95 962 American adults with a mean age of 65.4 years. In addition, compared with the 2007 WCRF/AICR guidelines used in the EPIC study, the 2018 guidelines we used removed the component “limit consumption of salt; avoid moldy cereals or pulses” and added the component “eat a diet rich in whole grains, vegetables, fruit, and beans.” Hence, the observed inconsistency may be also due to the difference in the components between two versions of the WCRF/AICR guidelines.

A hospital‐based case‐control study observed a significant inverse association of adherence to the 2007 WCRF/AICR guidelines with the risk of developing pancreatic cancer (odds ratio [OR] _≥5 vs <3.5_:0.41; 95% CI: 0.24, 0.68; *P*_trend_ = .0002),^11^ being consistent with our results. Interestingly, this case‐control study further observed that the benefits of adherence to the WCRF/AICR guidelines on pancreatic cancer risk were more pronounced in men (OR_≥5 vs <3.5_:0.28; 95% CI: 0.13, 0.63; *P*_trend_ = .0002) than in women (OR_≥5 vs <3.5_:0.64; 95% CI: 0.29, 1.43; *P*_trend_ = .1600).^11^ However, our study did not observe the modification effect of sex on the relevant results. Intuitively, this inconsistency may result from the differences in the study population. For instance, this case‐control^11^ study was conducted in an Italian population, and its proportion of males was obviously higher than that in our study (53.4% vs 48.7%). An alternative explanation is that this inconsistency is due to the differences in study design. It is well known that case‐control studies, especially hospital‐based ones, are more subject to reverse causation and selection bias than cohort studies. Moreover, they are prone to recall bias. Thus, the results from the above‐mentioned case‐control study^11^ might have been distorted by these inherent limitations. In fact, similar to our results, a number of observational studies on adherence to the WCRF/AICR guidelines and cancer risk did not detect the modification effect by sex.^9^, ^37^, ^38^, ^39^ Importantly, it should be reminded that the lack of significant interaction between the WCRF/AICR score and gender may also be attributable to the insufficient power, given a small number of outcome events involved in each subgroup. Hence, future studies with a larger sample size are needed to assess whether the association of the WCRF/AICR score with the risk of pancreatic cancer could be modified by sex.

Our study has several limitations. First, we did not consider all 2018 WCRF/AICR recommendations when constructing the WCRF/AICR scoring system, such as “do not use supplements for cancer prevention.” Thus, the beneficial effects of adherence to the WCRF/AICR guidelines could have been underestimated in our study. Second, our results may be susceptible to unrecognized or unmeasured confounders, although we have fully adjusted the potential confounders. However, this possibility appears to be relatively low, given that in our study setting, the *E*‐values were 2.35 and 2.45 for pancreatic cancer incidence and mortality, respectively. Moreover, this is an inherent limitation for any observational study. Third, in this study, daily food consumption used for the WCRF/AICR score construction was evaluated once at baseline. The evaluation of food consumption at one‐time point possibly leads to non‐differential bias, given that dietary habits may change over time. Nevertheless, in nutritional epidemiology, there is a classic assumption that an adult's dietary habits would not change substantially during several years^40^; it has also been indicated that the approach using a baseline diet only generally produces a weaker association than that using the cumulative averages.^41^

## 5 CONCLUSIONS

In the US population, adherence to the 2018 WCRF/AICR recommendations, especially “be physically active,” is associated with reduced risks of pancreatic cancer incidence and mortality. However, adherence to dietary recommendations alone does not confer such beneficial effects. Future studies should validate these findings in other populations and settings.

## CONFLICT OF INTEREST

None declared.

## AUTHORS' CONTRIBUTIONS

Guo‐Chao Zhong and Qu‐Jin Li conceived the study idea. Zhi‐Qing Zhang drafted the study protocol and initial manuscript. Fa‐Bao Hao made critical comments and revisions for the initial manuscript. Shan Liu was responsible for statistical analyses. Shan Liu and You‐Qi‐Le Wu interpreted the results of statistical analyses together. All authors approved the final version of the article, including the authorship list.

## Supporting information

Tables S1‐S7
Figures S1‐S4

## Data Availability

Original data involved in the present study are not freely available to the public because of the US National Cancer Institute's data policy. Nevertheless, these original data can be accessible upon the reasonable request and the final approval by the US National Cancer Institute.
