# Supplementary material for: Adherence to the 2018 World Cancer Research Fund/American Institute for Cancer Research cancer prevention recommendations and pancreatic cancer incidence and mortality: A prospective cohort study
Source: Cancer Med. 2020 Jul 27;9(18):6843–53. doi: 10.1002/cam4.3348 (PMC7520356; doi:10.1002/cam4.3348)
Supplement: Supplementary file 1 — Tables S1‐S7 Figures S1‐S4 [file CAM4-9-6843-s001.pdf]

SUPPLEMENTAL MATERIAL

Adherence to the 2018 World Cancer Research Fund/American Institute for Cancer Research cancer prevention recommendations and pancreatic cancer incidence and mortality: a prospective cohort study

Zhi-Qing Zhang, Qu-Jin Li, Fa-Bao Hao, You-Qi-Le Wu, Shan Liu, and Guo-Chao Zhong

Supplemental Table 1. The 2018 WCRF/AICR recommendations for cancer prevention and adherence score construction in the Prostate, Lung, Colorectal, and Ovarian Cancer Screening Trial

WCRF/AICR recommendations	Baseline data	Operationalization	Score	Number of participants (%)
Be a healthy weight	BMI (kg/m^2)	18.5–24.9	1	32350 (33.7)
		25.0–29.9	0.5	40977 (42.7)
		<18.5 or ≥30.0	0	22635 (23.6)
Be physically active	Moderate to vigorous physical activity (min/week)	≥150	1	31539 (32.9)
		75 to <150	0.5	20916 (21.8)
		<75	0	43507 (45.3)
Eat a diet rich in whole grains, vegetables, fruit, and beans	Fruits and vegetables (g/day)	≥400	1	62265 (64.9)
		200 to <400	0.5	26657 (27.8)
		<200	0	7040 (7.3)
	Dietary fiber (g/day)	≥30	1	8209 (8.6)
		15 to <30	0.5	48168 (50.2)
		<15	0	39585 (41.3)
Limit consumption of “fast foods” and other processed foods high in fat, starches or sugars	Ultra-processed foods (g/day)	Tertile 1: ≤142.9	1	31991 (33.4)
		Tertile 2: 143.0–233.9	0.5	31985 (33.3)
		Tertile 3: ≥234.0	0	31986 (34.8)
Limit consumption of red and processed meat	Red meat and processed meat (g/week)	Red meat <500 and processed meat <21	1	69636 (72.6)
		Red meat <500 and processed meat 21 to <100	0.5	25667 (26.7)
		Red meat ≥500 or processed meat ≥100	0	659 (0.7)
Limit consumption of sugar-sweetened drinks	Fruit or vegetable drinks and soft drinks (g/day)	0	1	11713 (12.2)
		>0 to ≤250	0.5	64780 (67.5)
		>250	0	19469 (20.3)

Limit alcohol consumption	Alcohol intake (g/day)	0	1	24171 (25.2)
		>0 to ≤28 for males and ≤14 for females	0.5	59666 (62.2)
		>28 for males and >14 for females	0	12125 (12.6)
For mothers: breastfeed your baby, if you can	Cumulative duration of exclusive breastfeeding (months)	≥6	1	15515 (16.2)
		<6	0.5	13399 (14.0)
		0	0	67048 (69.9)

Supplemental Table 2. Hazard ratios of the association between WCRF/AICR score and pancreatic cancer incidence in 77557 participants with complete data

Tertiles of WCRF/AICR score (range)	Number of cases	Person-years	Incidence/10000 person-years	Hazard ratio (95% confidence interval)		
				Unadjusted	Model 1[a]	Model 2 [b]
Overall WCRF/AICR score						
Tertile 1 (0.50–3.75)	91	167562.2	5.43	1.00 (reference)	1.00 (reference)	1.00 (reference)
Tertile 2 (4.00–4.75)	103	246089.1	4.19	0.77 (0.58, 1.02)	0.76 (0.57, 1.02)	0.76 (0.57, 1.02)
Tertile 3 (5.00–8.00)	83	277480.1	2.99	0.55 (0.41, 0.74)	0.56 (0.40, 0.78)	0.56 (0.40, 0.77)
P_{trend}				<0.0001	0.0005	0.0005
Continuous (1-ponit increment)	277	691131.4	4.01	0.81 (0.72, 0.91)	0.81 (0.71, 0.93)	0.81 (0.71, 0.93)
Dietary WCRF/AICR score						
Tertile 1 (0.50–2.75)	86	196342.0	4.38	1.00 (reference)	1.00 (reference)	1.00 (reference)
Tertile 2 (3.00–3.25)	82	189702.3	4.32	0.99 (0.73, 1.33)	0.95 (0.70, 1.30)	0.96 (0.70, 1.31)
Tertile 3 (3.50–5.00)	109	305087.1	3.57	0.82 (0.61, 1.08)	0.77 (0.56, 1.05)	0.78 (0.57, 1.06)
P_{trend}				0.1245	0.0800	0.0906
Continuous (1-ponit increment)	277	691131.4	4.01	0.86 (0.72, 1.03)	0.82 (0.68, 1.00)	0.83 (0.68, 1.01)

Abbreviation: WCRF/AICR, World Cancer Research Fund and American Institute for Cancer Research.

[a] Adjusted for variables determined by change-in-estimate strategy, namely age (years), sex (male, female), smoking status (current, former, never), history of diabetes (yes, no), and total energy intake (kcal/day).

[b] Adjusted for age (years), sex (male, female), race (non-Hispanic white, non-Hispanic black, Hispanic, others), educational degree (college below, college graduate, postgraduate), smoking status (current, former, never), aspirin use (yes, no), history of diabetes (yes, no), family history of pancreatic cancer (yes, no), and total energy intake (kcal/day). For the association of dietary WCRF/AICR score with pancreatic cancer incidence, model 2 was further adjusted for body mass index (kg/m^2) and moderate to vigorous activity (min/week).

Supplemental Table 3. Sensitivity analyses on the association between WCRF/AICR score and pancreatic cancer incidence

Tertiles of WCRF/AICR score (range)	Hazard ratio (95% confidence interval) [a]	
	Excluding cases occurring within the first two years of follow-up (n=58)	Excluding subjects with extreme values of energy intake (n=2684) [b]
Overall WCRF/AICR score		
Tertile 1	1.00 (reference)	1.00 (reference)
Tertile 2	0.96 (0.72, 1.28)	0.90 (0.69, 1.16)
Tertile 3	0.72 (0.51, 1.00)	0.67 (0.49, 0.91)
P_{trend}	0.0626	0.0125
Continuous (1-ponit increment)	0.90 (0.80, 1.03)	0.87 (0.78, 0.98)
Dietary WCRF/AICR score		
Tertile 1	1.00 (reference)	1.00 (reference)
Tertile 2	1.14 (0.84, 1.55)	1.04 (0.78, 1.38)
Tertile 3	0.86 (0.63, 1.19)	0.84 (0.62, 1.12)
P_{trend}	0.2228	0.1641
Continuous (1-ponit increment)	0.87 (0.71, 1.06)	0.85 (0.71, 1.02)

Abbreviation: WCRF/AICR, World Cancer Research Fund and American Institute for Cancer Research.

[a] Adjusted for age (years), sex (male, female), race (non-Hispanic white, non-Hispanic black, Hispanic, others), educational level (college below, college graduate, postgraduate), smoking status (current, former, never), aspirin use (yes, no), history of diabetes (yes, no), family history of pancreatic cancer (yes, no), and total energy intake (kcal/day). For the association of dietary WCRF/AICR score with pancreatic cancer incidence, model 2 was further adjusted for body mass index (kg/m^2) and moderate to vigorous activity (min/week).

[b] Extreme values of energy intake are defined as <800 or >4000 kcal/d for men and <500 or >3500 kcal/d for women.

Supplemental Table 4. Associations of adherence to individual WCRF/AICR cancer prevention recommendations with pancreatic cancer incidence

WCRF/AICR components	Number of cases	Person-years	Hazard ratio (95% confidence interval) [a]	P_{trend}
Be a healthy weight				
0	82	196471.8	1.00 (reference)	0.8146
0.5	143	364285.5	0.91 (0.69, 1.20)	
1	112	289973.4	0.96 (0.71, 1.29)	
Be physically active				
0	179	382252.5	1.00 (reference)	0.0377
0.5	71	186319.3	0.91 (0.69, 1.20)	
1	87	282158.9	0.75 (0.58, 0.98)	
Eat a diet rich in whole grains, vegetables, fruit, and beans				
0	27	56980.9	1.00 (reference)	0.6664
0.25	70	180441.1	0.88 (0.56, 1.38)	
0.5	65	169194.1	0.90 (0.56, 1.42)	
0.75	150	372377.8	1.01 (0.64, 1.58)	
1	25	71736.8	0.95 (0.49, 1.83)	
Limit consumption of ultra-processed foods				
0	120	284464.0	1.00 (reference)	0.7891
0.5	102	284794.4	0.84 (0.62, 1.14)	
1	115	281472.3	0.94 (0.66, 1.34)	
Limit consumption of red and processed meat				
0	6	5700.4	1.00 (reference)	0.2827
0.5	96	225612.9	0.39 (0.17, 0.90)	
1	235	619417.4	0.37 (0.16, 0.86)	
Limit consumption of sugar-sweetened drinks				
0	76	171716.6	1.00 (reference)	0.0854
0.5	227	575246.4	0.88 (0.67, 1.15)	
1	34	103767.7	0.69 (0.45, 1.05)	
Limit alcohol consumption				
0	46	107670.0	1.00 (reference)	0.4144
0.5	205	530430.7	0.85 (0.61, 1.19)	
1	86	212630.0	0.83 (0.56, 1.22)	
For mothers: breastfeed your baby, if you can				
0	57	181897.0	1.00 (reference)	0.7975
0.5	47	120323.0	1.18 (0.80, 1.74)	
1	41	138539.5	0.93 (0.62, 1.40)	

[a] Adjusted for age (years), sex (male, female) (except the component "for mothers: breastfeed your baby, if you can"), race (non-Hispanic white, non-Hispanic black, Hispanic, others), educational degree (college below, college graduate, postgraduate), smoking status (current, former, never), aspirin use (yes, no), history of diabetes (yes, no), family history of pancreatic cancer (yes, no), and total energy intake (kcal/day). All individual components were mutually adjusted.

Supplemental Table 5. Subdistribution hazard ratios of the association of WCRF/AICR score with pancreatic cancer mortality in 77557 participants with complete data.

Tertiles of WCRF/AICR score (range)	Number of deaths	Person-years	Mortality/10000 person-years	Subdistribution hazard ratio (95% confidence interval)		
				Unadjusted	Model 1[a]	Model 2 [b]
Overall WCRF/AICR score						
Tertile 1 (0.50–3.75)	80	249068.2	3.21	1.00 (reference)	1.00 (reference)	1.00 (reference)
Tertile 2 (4.00–4.75)	97	369617.8	2.62	0.82 (0.61, 1.11)	0.81 (0.60, 1.10)	0.81 (0.60, 1.10)
Tertile 3 (5.00–8.00)	75	421666.4	1.78	0.56 (0.41, 0.77)	0.56 (0.39, 0.80)	0.56 (0.39, 0.80)
P_{trend}				0.0003	0.0011	0.0011
Continuous (1-ponit increment)	252	1040352.3	2.42	0.83 (0.73, 0.93)	0.83 (0.72, 0.95)	0.83 (0.72, 0.95)
Dietary WCRF/AICR score						
Tertile 1 (0.50–2.75)	75	295609.4	2.54	1.00 (reference)	1.00 (reference)	1.00 (reference)
Tertile 2 (3.00–3.25)	75	285784.8	2.62	1.04 (0.75, 1.43)	1.00 (0.72, 1.39)	1.01 (0.72, 1.40)
Tertile 3 (3.50–5.00)	102	458958.2	2.22	0.87 (0.65, 1.18)	0.82 (0.59, 1.14)	0.82 (0.59, 1.15)
P_{trend}				0.3018	0.1887	0.1916
Continuous (1-ponit increment)	252	1040352.3	2.42	0.89 (0.74, 1.07)	0.85 (0.69, 1.04)	0.84 (0.69, 1.04)

Abbreviation: WCRF/AICR, World Cancer Research Fund and American Institute for Cancer Research.

[a] Adjusted for variables determined by change-in-estimate strategy, namely age (years), sex (male, female), smoking status (current, former, never), history of diabetes (yes, no), and total energy intake (kcal/day).

[b] Adjusted for age (years), sex (male, female), race (non-Hispanic white, non-Hispanic black, Hispanic, others), educational level (college below, college graduate, postgraduate), smoking status (current, former, never), aspirin use (yes, no), history of diabetes (yes, no), family history of pancreatic cancer (yes, no), and total energy intake (kcal/day). For the association of dietary WCRF/AICR score with pancreatic cancer mortality, model 2 was further adjusted for body mass index (kg/m^2) and moderate to vigorous activity (min/week).

Supplemental Table 6. Sensitivity analyses on the association between WCRF/AICR score and pancreatic cancer mortality

Tertiles of WCRF/AICR score (range)	Subdistribution hazard ratio (95% confidence interval) [a]	
	Excluding deaths occurring within the first two years of follow-up (n=38)	Excluding subjects with extreme values of energy intake (n=2684) [b]
Overall WCRF/AICR score		
Tertile 1	1.00 (reference)	1.00 (reference)
Tertile 2	1.01 (0.76, 1.34)	0.94 (0.72, 1.24)
Tertile 3	0.66 (0.47, 0.94)	0.65 (0.47, 0.90)
P_{trend}	0.0308	0.0134
Continuous (1-ponit increment)	0.89 (0.78, 1.01)	0.87 (0.77, 0.99)
Dietary WCRF/AICR score		
Tertile 1	1.00 (reference)	1.00 (reference)
Tertile 2	1.14 (0.83, 1.56)	1.05 (0.78, 1.42)
Tertile 3	0.84 (0.61, 1.17)	0.85 (0.62, 1.15)
P_{trend}	0.2451	0.2027
Continuous (1-ponit increment)	0.85 (0.69, 1.04)	0.84 (0.70, 1.02)

Abbreviation: WCRF/AICR, World Cancer Research Fund and American Institute for Cancer Research.

[a] Adjusted for age (years), sex (male, female), race (non-Hispanic white, non-Hispanic black, Hispanic, others), educational level (college below, college graduate, postgraduate), smoking status (current, former, never), aspirin use (yes, no), history of diabetes (yes, no), family history of pancreatic cancer (yes, no), and total energy intake (kcal/day). For the association of dietary WCRF/AICR score with pancreatic cancer mortality, model 2 was further adjusted for body mass index (kg/m2) and moderate to vigorous activity (min/week).

[b] Extreme values of energy intake are defined as <800 or >4000 kcal/d for men and <500 or >3500 kcal/d for women.

Supplemental Table 7. Associations of adherence to individual WCRF/AICR cancer prevention recommendations with pancreatic cancer mortality

WCRF/AICR components	Number of deaths	Person-years	Subdistribution hazard ratio (95% confidence interval) [a]	P_{trend}
Be a healthy weight				
0	76	294707.7	1.00 (reference)	0.7576
0.5	130	547862.5	0.90 (0.68, 1.21)	
1	101	437178.3	0.94 (0.69, 1.29)	
Be physically active				
0	162	565417.0	1.00 (reference)	0.0542
0.5	64	282481.4	0.91 (0.67, 1.21)	
1	81	431850.1	0.77 (0.58, 1.01)	
Eat a diet rich in whole grains, vegetables, fruit, and beans				
0	25	85570.7	1.00 (reference)	0.5663
0.25	60	271174.9	0.82 (0.51, 1.32)	
0.5	61	253982.9	0.92 (0.57, 1.48)	
0.75	142	561065.9	1.04 (0.65, 1.66)	
1	19	107954.1	0.76 (0.37, 1.55)	
Limit consumption of ultra-processed foods				
0	108	427847.6	1.00 (reference)	0.8350
0.5	90	428087.2	0.84 (0.61, 1.16)	
1	109	423813.6	1.02 (0.70, 1.48)	
Limit consumption of red and processed meat				
0	6	8507.7	1.00 (reference)	0.1935
0.5	88	337777.7	0.36 (0.15, 0.84)	
1	213	933463.1	0.33 (0.14, 0.78)	
Limit consumption of sugar-sweetened drinks				
0	70	258937.7	1.00 (reference)	0.1010
0.5	205	865460.8	0.86 (0.64, 1.13)	
1	32	155350.0	0.70 (0.45, 1.08)	
Limit alcohol consumption				
0	42	161990.0	1.00 (reference)	0.2955
0.5	189	803104.4	0.86 (0.60, 1.22)	
1	76	314654.1	0.80 (0.53, 1.20)	
For mothers: breastfeed your baby, if you can				
0	54	275890.6	1.00 (reference)	0.8152
0.5	42	181824.4	1.12 (0.74, 1.67)	
1	39	211517.7	0.94 (0.62, 1.43)	

[a] Adjustment for age (years), sex (male, female) (except the component "for mothers: breastfeed your baby, if you can"), race (non-Hispanic white, non-Hispanic black, Hispanic, others), educational level (college below, college graduate, postgraduate), smoking status (current, former, never), aspirin use (yes, no), history of diabetes (yes, no), family history of pancreatic cancer (yes, no), and energy intake from diet (kcal/day). All WCRF/AICR components were mutually adjusted.

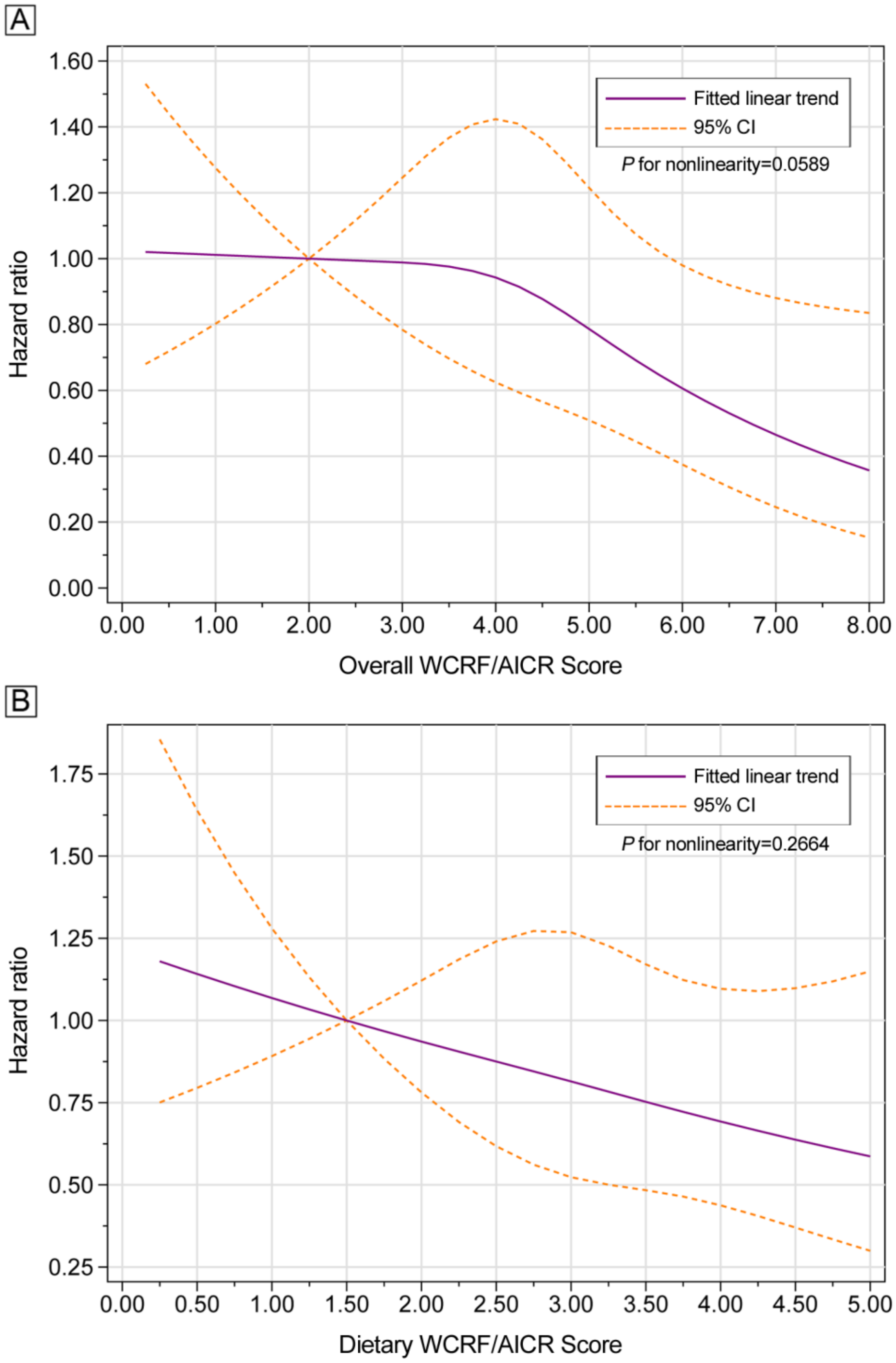

Supplemental Figure 1 Dose–response analyses on WCRF/AICR scores and pancreatic cancer incidence. The referents were set as 2.00 and 1.50 for overall and dietary WCRF/AICR scores, respectively. Hazard ratio was adjusted for age, sex, race, educational degree, smoking status, aspirin use, history of diabetes, family history of pancreatic cancer, and energy intake from diet. For the dose–response analysis on the dietary WCRF/AICR score, hazard ratio was further adjusted for body mass index and physical activity level. The purple solid line represents the fitted linear trend, and the orange short-dash line represents 95% CI. WCRF/AICR, World Cancer Research Fund/American Institute for Cancer Research; CI, confidence interval.

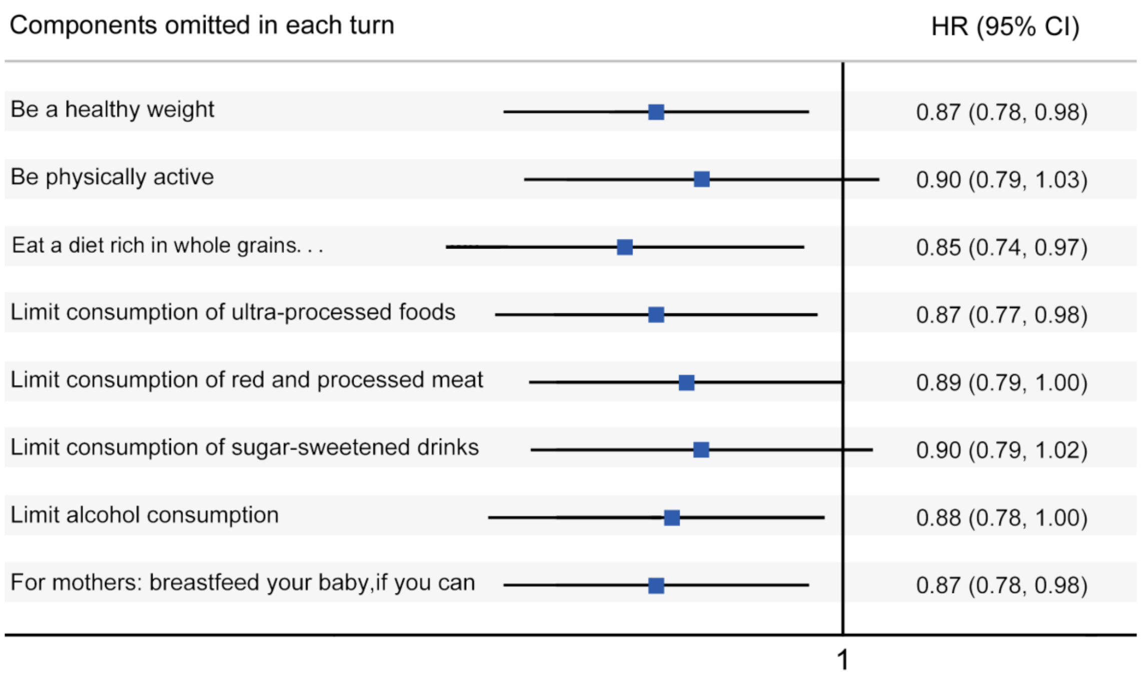

Supplemental Figure 2 Explanatory analysis: ignoring a single component in turn to determine their relative importance in the overall WCRF/AICR score. The component shown on the left is the one left out in each turn. The square with dark blue color represents the HR after ignoring a single component, and the corresponding black line represents its 95% CI. HR represents risk estimate per 1-point increment in the overall WCRF/AICR score, and was adjusted for age, sex, race, educational degree, smoking status, aspirin use, history of diabetes, family history of pancreatic cancer, and energy intake from diet. All WCRF/AICR components were mutually adjusted. WCRF/AICR, World Cancer Research Fund/American Institute for Cancer Research; HR, hazard ratio; CI, confidence interval.

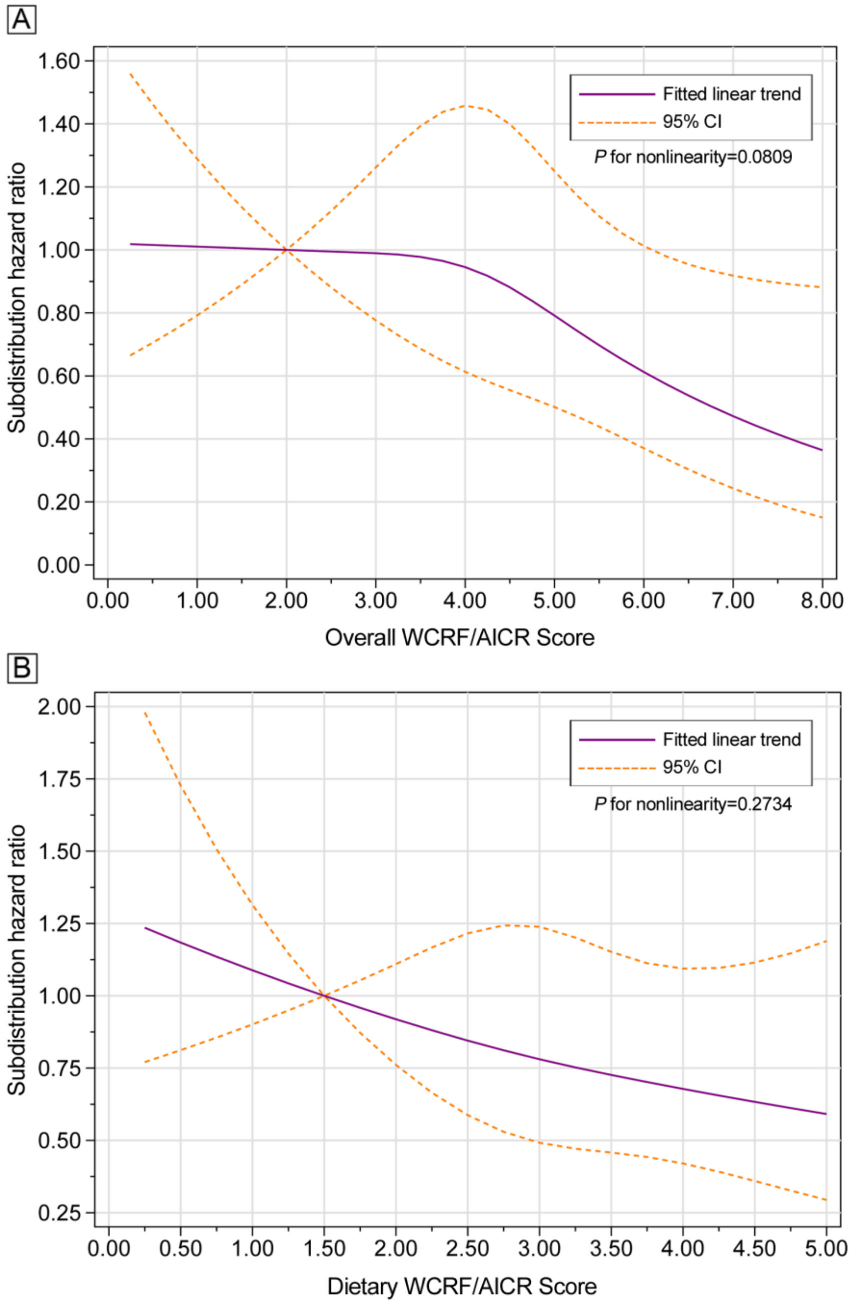

Supplemental Figure 3 Dose–response analyses on WCRF/AICR scores and pancreatic cancer mortality. The referents were set as 2.00 and 1.50 for overall and dietary WCRF/AICR scores, respectively. Subdistribution hazard ratio was adjusted for age, sex, race, educational degree, smoking status, aspirin use, history of diabetes, family history of pancreatic cancer, and energy intake from diet. For the dose–response analysis on the dietary WCRF/AICR score, subdistribution hazard ratio was further adjusted for body mass index and physical activity level. The purple solid line represents the fitted linear trend, and the orange short-dash line represents 95% CI. WCRF/AICR, World Cancer Research Fund/American Institute for Cancer Research; CI, confidence interval.

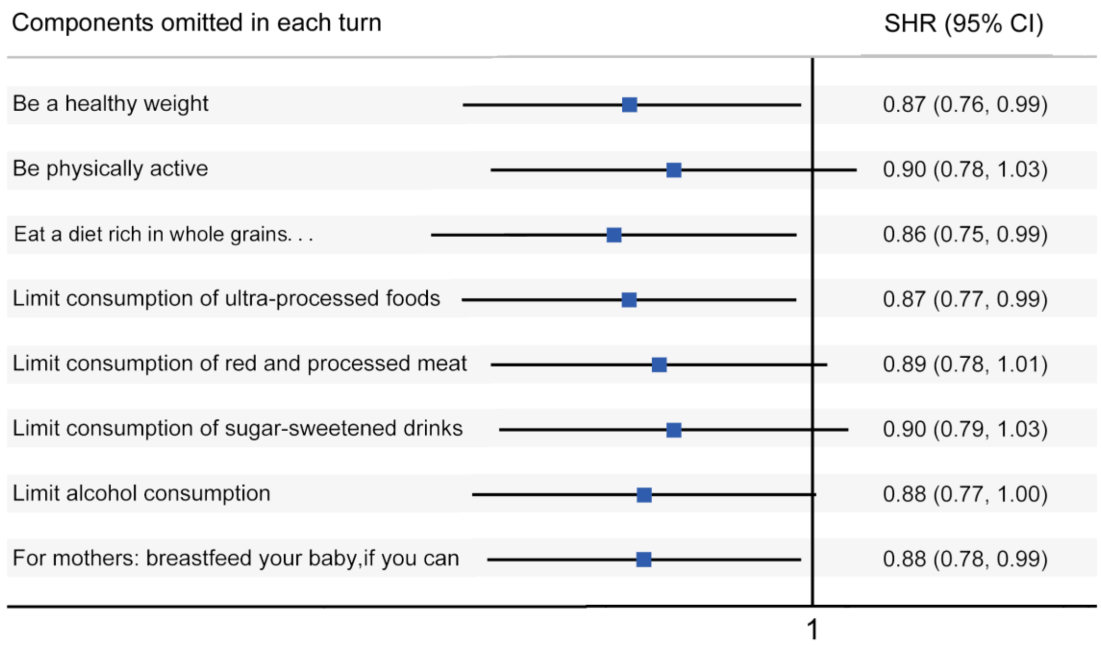

Supplemental Figure 4 Explanatory analysis: ignoring a single component in turn to determine their relative importance in the overall WCRF/AICR score. The component shown on the left is the one left out in each turn. The square with dark blue color represents the SHR after ignoring a single component, and the corresponding black line represents its 95% CI. SHR represents risk estimate per 1-point increment in the overall WCRF/AICR score, and was adjusted for age, sex, race, educational degree, smoking status, aspirin use, history of diabetes, family history of pancreatic cancer, and energy intake from diet. All WCRF/AICR components were mutually adjusted. WCRF/AICR, World Cancer Research Fund/American Institute for Cancer Research; SHR, subdistribution hazard ratio; CI, confidence interval.
